# New markers for human ovarian cancer that link platinum resistance to the cancer stem cell phenotype and define new therapeutic combinations and diagnostic tools

**DOI:** 10.1186/s13046-019-1245-5

**Published:** 2019-06-03

**Authors:** Sandra Muñoz-Galván, Blanca Felipe-Abrio, Miguel García-Carrasco, Julia Domínguez-Piñol, Elisa Suarez-Martinez, Eva M. Verdugo-Sivianes, Asunción Espinosa-Sánchez, Lola E. Navas, Daniel Otero-Albiol, Juan J. Marin, Manuel P. Jiménez-García, Jose M. García-Heredia, Adoración G. Quiroga, Purificacion Estevez-Garcia, Amancio Carnero

**Affiliations:** 1 Instituto de Biomedicina de Sevilla, IBIS, Campus Hospital Universitario Virgen del Rocío, Universidad de Sevilla-Consejo Superior de Investigaciones Científicas, Avda. Manuel Siurot s/n, Seville, Spain; 2 CIBER de CANCER, Institute of Health Carlos III, Madrid, Spain; 3 Medical Oncology Unit, Hospital Universitario Virgen del Rocío, Seville, Spain; 4 Department of Vegetal Biochemistry and Molecular Biology, University of Seville, Seville, Spain; 5 Organic Chemistry Department, Autonomous University of Madrid, Madrid, Spain

**Keywords:** Ovarian cancer, Cancer stem cells, Biomarkers, Therapy

## Abstract

**Background:**

Ovarian cancer is the leading cause of gynecologic cancer-related death, due in part to a late diagnosis and a high rate of recurrence. Primary and acquired platinum resistance is related to a low response probability to subsequent lines of treatment and to a poor survival. Therefore, a comprehensive understanding of the mechanisms that drive platinum resistance is urgently needed.

**Methods:**

We used bioinformatics analysis of public databases and RT-qPCR to quantitate the relative gene expression profiles of ovarian tumors. Many of the dysregulated genes were cancer stem cell (CSC) factors, and we analyzed its relation to therapeutic resistance in human primary tumors. We also performed clustering and in vitro analyses of therapy cytotoxicity in tumorspheres.

**Results:**

Using bioinformatics analysis, we identified transcriptional targets that are common endpoints of genetic alterations linked to platinum resistance in ovarian tumors. Most of these genes are grouped into 4 main clusters related to the CSC phenotype, including the DNA damage, Notch and C-KIT/MAPK/MEK pathways. The relative expression of these genes, either alone or in combination, is related to prognosis and provide a connection between platinum resistance and the CSC phenotype. However, the expression of the CSC-related markers was heterogeneous in the resistant tumors, most likely because there were different CSC pools. Furthermore, our in vitro results showed that the inhibition of the CSC-related targets lying at the intersection of the DNA damage, Notch and C-KIT/MAPK/MEK pathways sensitize CSC-enriched tumorspheres to platinum therapies, suggesting a new option for the treatment of patients with platinum-resistant ovarian cancer.

**Conclusions:**

The current study presents a new approach to target the physiology of resistant ovarian tumor cells through the identification of core biomarkers. We hypothesize that the identified mutations confer platinum resistance by converging to activate a few pathways and to induce the expression of a few common, measurable and targetable essential genes. These pathways include the DNA damage, Notch and C-KIT/MAPK/MEK pathways. Finally, the combined inhibition of one of these pathways with platinum treatment increases the sensitivity of CSC-enriched tumorspheres to low doses of platinum, suggesting a new treatment for ovarian cancer.

**Electronic supplementary material:**

The online version of this article (10.1186/s13046-019-1245-5) contains supplementary material, which is available to authorized users.

## Background

Ovarian cancer (OC) is the seventh most frequent malignant tumor type in women worldwide and is the leading cause of death from gynecological cancer, accounting for 4% of cancer-related deaths (GLOBOCAN) [1]. The majority of patients are diagnosed at an advanced stage due to unspecific clinical manifestations. There are 4 main histologic subtypes that have been described: serous (approximately 70%), endometrioid, clear cell and mucinous. In recent years, evidence has shown that there are unique molecular features, treatment responses and prognoses for each of the subtypes. Genetic alterations involving DNA homologous recombination repair system (BRCA1/2, genes of the Fanconi anemia and DNA mismatch repair pathways) are the most investigated and have been identified in more than 30% of OCs. Other relevant alterations include defective Notch, PI3K, RAS-MEK and forkhead box protein M1 (*FOXM1*) signaling pathways, as well as mutations in *TP53, MTOR* or *MYC* in high-grade serous or endometrioid OCs, mutations in *ARID1A*, *PIK3CA* and *PTEN* in clear-cell carcinomas, and *KRAS*, *BRAF* or *CDKN2A* mutations in mucinous carcinomas [2].

Complete cytoreductive surgery that achieves the resection of all macroscopically visible disease is a major factor that determines the chances of success in the treatment of OC. Chemotherapy is always given after surgery since most of the patients will eventually relapse, except in cases of nonaggressive tumors and in very early stage tumors. Platinum agents constitute the most active group of chemotherapy drugs in ovarian cancer, and over the last decades, multiple studies have progressively optimized the efficacy and tolerability of the treatment. Combination schemes of cisplatin and taxanes demonstrated a higher survival benefit over monotherapy and other combinations, and the cisplatin analogue carboplatin confirmed similar efficacy and substantially better tolerance than cisplatin. Therefore, intravenous carboplatin in combination with paclitaxel every 3 weeks constitute the standard first-line treatment for OC [3]. Pegylated liposomal doxorubicin [4, 5] or docetaxel [6] are alternatives for patients who are not candidates for paclitaxel, and these treatments showed similar efficacy with a different toxicity profile. More recently, targeted therapies directed against angiogenesis (bevacizumab) and PARP inhibitors have demonstrated benefit in ovarian cancer, expanding available therapeutic options [2].

The clinical response rates to these drugs regularly exceed 60%, and the median time to the onset of recurrence usually exceeds 1 year even in the subset of women with suboptimal cytoreduction [2, 7–9]. In spite of surgery and chemotherapy administration, approximately 80% of the patients will relapse. Recurrent disease is generally incurable, and it is classified as platinum-resistant (recurrence < 6 months after last platinum dose) or platinum-sensitive (> 6 months). Platinum-resistant o sensitive status is one of the most important prognostic factors in recurrent disease and it is also a predictive factor of response of retreatment with platinum-based schemes. Platinum-resistant tumors show dismal outcomes with median overall survival less than 12 months [3]. Therefore, the search for new compounds that may be active in platinum-resistant tumors (primary or acquired after treatment) is a necessity for these patients. In addition, the identification of platinum response biomarkers would help to discriminate patients, avoiding the administration of high doses of cytotoxic compounds to patients who would not obtain a real benefit.

Many mutations have been found to be responsible for the resistance to platinum drugs (TCGA, [7, 10–15]), although their complexity makes the analysis of ovarian cancer resistance difficult. We hypothesize that the many known mutations that confer platinum resistance are distributed among different pathways, which may activate a few common essential effector genes. Ultimately, these effector genes may be responsible for the “ovarian cancer resistance physiology”, which may be measurable, predictive and targetable.

In this study, we performed a bioinformatic analysis with public databases to analyze transcriptional alterations that were common in ovarian tumors, mainly linked to recurrence. It was hypothesized that these alterations were causally connected to resistance to platinum therapy. After individual validation, the data suggest that these genetic alterations are involved in the acquisition of stemness properties that are linked to the resistance to therapy in ovarian tumor cells. Finally, the inhibition of key regulators of the stemness phenotype can recover sensitivity to platinum in stem cell surrogate assays.

## Methods

### Study approval

Written informed consent was provided by all patients. This project was approved by the Research Ethics Committee of the Hospital Universitario Virgen del Rocio (CEI 0309-N-15). All tissue samples and patient information were treated in accordance with the Declaration of Helsinki.

### Patient cohort

A cohort of paraffin-embedded tissue samples from 21 patients with ovarian cancer were obtained from the biobank of the Hospital Universitario Virgen del Rocío-Instituto de Biomedicina de Sevilla (Sevilla, Spain) for RNA expression studies and for a correlation analysis of the clinicopathological features. Samples were obtained from biopsies of patients subjected to platinum treatment who were evaluated for their response according to the RECIST criteria, and normal tissue, platinum-resistant and platinum-sensitive tumor samples were obtained. Tumor samples were sent to the pathology laboratory for diagnosis and were prepared for storage with formalin fixation and paraffin embedding. Samples were stained with hematoxylin/eosin, and RNA was extracted and obtained from tumor tissue.

### Public databases of clinical samples

To validate our results, we obtained data from publicly available clinical and genomic databases, including Oncomine (https://powertools.oncomine.com/) and the TCGA Research Network (http://cancergenome.nih.gov/).

### RT–qPCR

Total RNA from paraffin-embedded tissue samples was purified using a Recover All Total Nucleic acid isolation Kit (Invitrogen) according to the manufacturer’s instructions, but with slight modifications; specifically, digestion was performed for 3 h at 50 °C and 15 min at 80 °C. Total RNA from tumorspheres and total adherent cultured cells (total culture samples) was purified using a ReliaPrepTM RNA Tissue Miniprep System (Promega, Fitchburg, WI, USA) according to the manufacturer’s instructions. Reverse transcription was performed with 0.5 μg of mRNA using a High-Capacity cDNA Reverse Transcription C-KIT (Life Technologies) according to the manufacturer’s recommendations. The PCR mixture (10 μl) contained 2 μl of the reverse transcription reaction product diluted 1:6, 2.5 μl of water, 5 μl of GoTaqR Probe qPCR Master Mix (Promega) and 0.5 μl of the appropriate TaqMan Assay (20X) containing primers and a probe for the mRNA of interest (Applied Biosystems). We used the following probes (Applied Biosystems): ADRB3 (Hs_00609046_m1), ANG (Hs04195574_s1), BTG2 (Hs00198887_m1), ESD (Hs00382667_m1), FBXL7 (Hs00202348_m1), RAD51 (Hs00947967_m1), ST13 (Hs00832556_s1), ST7L (Hs00373316_m1), DUSP4 (Hs01027785_m1), AP1M2 (Hs01091817_m1), CKAP4 (Hs_00199135_m1), C-KIT (Hs00174029_m1), DUSP1 (Hs00610256_g1), PAX8 (Hs01015257_g1), NOTCH3 (Hs01128541_m1), CD133 (Hs01009257_m1), NANOG (Hs04260366_g1), CXCR4 (Hs00607978_s1), ABCG2 (Hs01053790_m1) and GAPDH (Hs03929097_g1). We analyzed the quality of RNA obtained from the tumor samples and normalized expression levels to the housekeeping gene *GAPDH.*

### Cell culture

Cells were cultured according to the manufacturer’s recommended procedure. Briefly, SKOV3 and OVCAR8 were cultured in RPMI and incubated at 37 °C with 5% CO_2_ in a humidified atmosphere.

### Tumorsphere assay

Cells were washed once with PBS and then harvested with 0.025% trypsin-EDTA. A total of 5 × 10^3^ cells of each cell line were resuspended in 1 ml of complete MammoCult medium (contains the MammoCult Basal medium, MammoCult Proliferation Supplement, fresh hydrocortisone and heparin; STEMCELL Technologies) and seeded in ultralow attachment 24-well plates (Corning #3473). Cultures were incubated in a 5% CO_2_ humidified incubator at 37 °C for 4 days. Tumorspheres were then visualized by inverted microscopy (Olympus IX-71) and were counted. Experiments were independently repeated a minimum of three times in triplicate.

### Cytotoxic MTT assay

A total of 5 × 10^3^ SKOV3 or OVCAR8 cells were seeded to form tumorspheres and then treated 24 h later with platinum drugs or/and Notch or C-KIT inhibitors (DATP or imatinib, respectively). After 96 h, the cell viability was measured with MTT.

### Quantification and statistical analysis

All statistical analyses were performed using GraphPad Prism 4. The distribution of quantitative variables among different study groups was assessed using parametric (Student’s *t*-test) or nonparametric (Kruskal–Wallis or Mann–Whitney) tests, as appropriate. Experiments were performed a minimum of three times and were always performed as independent triplicates. Survival data from the patient databases were analyzed with the log-rank Mantel-Cox statistical test.

### Analyses of cancer patient databases

We performed meta-analyses of the PrognoScan public patient datasets (http://dna00.bio.kyutech.ac.jp/PrognoScan/) to analyze the expression levels in tumor and non-tumor databases for ovary tissue samples. Statistical significance versus normal samples was considered to be *P* < 0.05. Patient survival was analyzed using the R2 Genomics analysis and visualization platform (http://hgserver1.amc.nl), developed by the Department of Oncogenomics of the Academic Medical Center (AMC) (Amsterdam, Netherlands). Kaplan-Meier plots showing patient survival were generated for databases with available survival data using the scan method, which search for the optimum survival cut-off based on statistical analyses (log-rank test), thus finding the most significant expression cut-off. To analyze the protein network, we use the web portal https://string-db.org.

## Results

### Identification of biomarkers for poor prognosis in ovarian tumors

We performed an analysis of publicly available transcriptional datasets (Additional file 1: Table S1) to identify genes whose expression may be highly deregulated (levels of transcription > 4-fold higher or < − 4-fold lower than the mean of the non-tumor samples) in ovarian tumors. In these datasets, the most represented tumor type was serous adenocarcinoma, although different ovarian tumor subtypes were discriminated in different datasets. We compared the mRNA levels in tumors with the mRNA levels in normal ovarian tissue and found genes that were up- or down-regulated with clear statistical significance (*P* < 0.01) (Table 1). Among the upregulated genes in the tumors compared to those in the normal tissue, we found *CKAP4*, *DUSP1*, *PAX8*, *AP1M2*, *C-KIT* and *NOTCH3*, with *NOTCH3* being the only gene that was upregulated in all tumor types. *PAX8* and *C-KIT* were upregulated in most tumor types, while *CKAP4* and *DUSP1* were upregulated only in serous and mucinous adenocarcinoma. Interestingly, *AP1M2* was highly upregulated exclusively in clear cell carcinoma compared to normal tissue. On the other hand, 9 genes were found to be downregulated in tumor vs nontumor tissue (Table 1). *ST13* was commonly downregulated in all tumor types compared to normal tissue. Compared to the expression levels in normal tissue, other genes, such as *ADRB3*, *BTG2*, *DUSP4*, *RAD51* or *FBLX7,* were downregulated in specific tumor types, and *ANG*, *ESD* and *STL7* were downregulated in most tumor types. In general, the highly deregulated genes in ovarian tumors have a very wide range of functions, families and chromosomal loci.

**Table 1:** Upregulated or downregulated genes found in ovary tumors vs non-tumoral ovary tissue

Serous Adc	Mucinous	Endometroid	Carcinoma	Clear Cell C.	Gene definition
Upregulated					
CKAP4	CKAP4				Cytoskeleton associated protein 4
DUSP1	DUSP1				Dual specificity phosph atase 1
				AP1M2	Adaptor-rela ted protein complex1, Mu2 subunit
PAX8		PAX8	PAX8	PAX8	Paired box 8
NOTCH3	NOTCH3	NOTCH3	NOTCH3	NOTCH3	Notch homolog 3
C-KIT	C-KIT			C-KIT	Mast cell growth factor receptor
Downegulated					
ADRB3	ADRB3				Adrenergic B3 receptor
ANG		ANG	ANG	ANG	Angiogenin
BTG2					BTG family member 2
	DUSP4	DUSP4			Dual specifi city phosph atase 4
	FBXL7			FBXL7	F-Box and leucin rich rep eat protein 7
ESD	ESD	ESD		ESD	Esterase D
ST13	ST13	ST13	ST13	ST13	Suppressor of tumors 13
ST7L		ST7L		ST7L	Suppressor of tumors 7 like
RAD51					BRCA1/BRCA2-containing complex, subunit 5

Next, we analyzed the mutational profile of these genes in ovarian cancer using the TCGA database on cBioportal. We did not observe any clear mutational profile among those genes, except for *C-KIT* (Additional file 1: Table S2). However, most of the genes showed clear chromosomal alterations (either amplification or deletions) and clear mRNA alterations (Additional file 1: Table S2). In general, these alterations correlated well with the observed pattern of behavior found in ovarian tumors. The analysis of promoter methylation indicated that most genes showed a methylation pattern that was compatible with the expression data (Additional file 1: Figure S1).

**Fig. 1:**
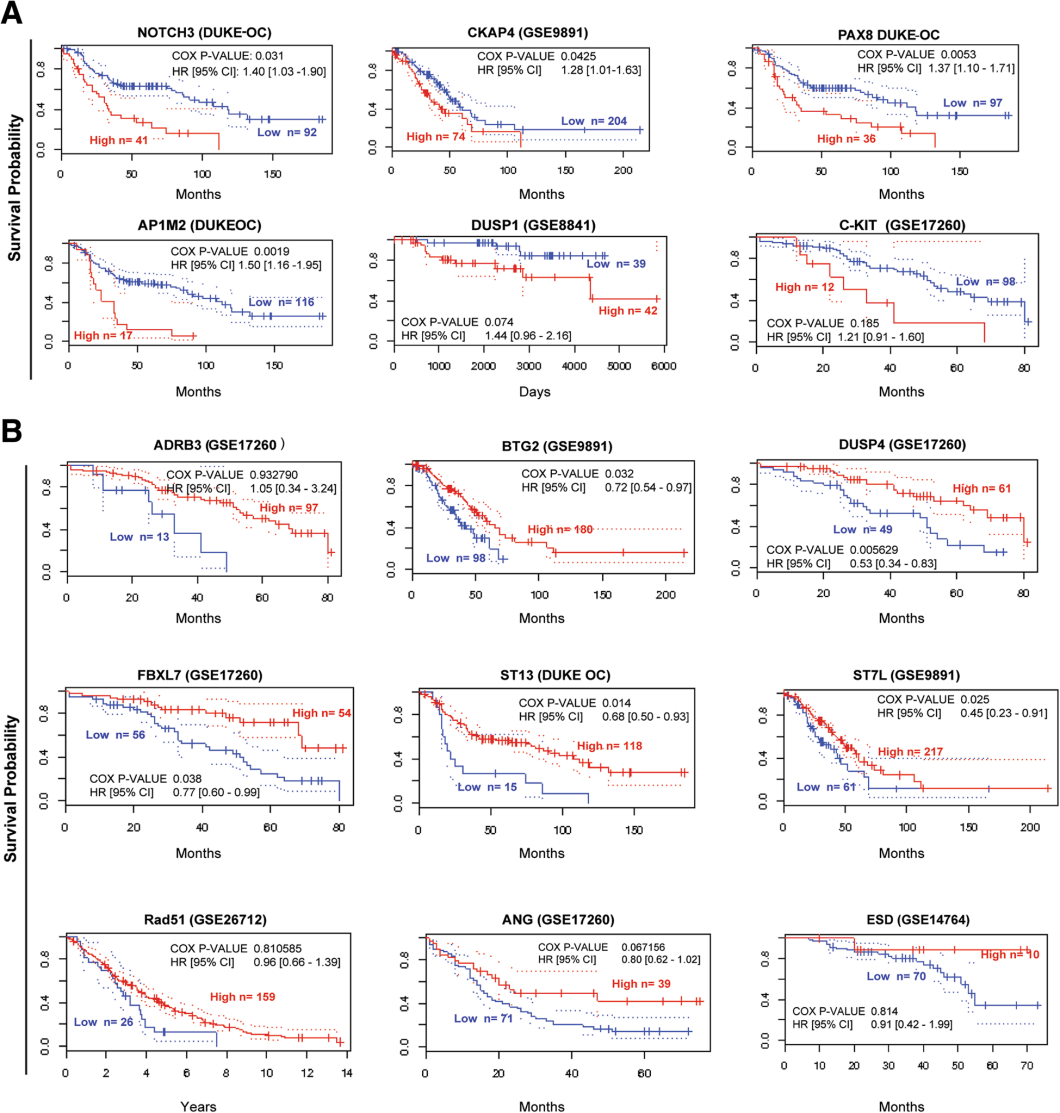
Analysis of the survival probability of ovarian cancer patients in different datasets by the expression of the identified genes. Cox analysis of survival probability of patients according to mRNA levels of different genes. Kaplan-Meier curves of the genes up-regulated (**a**) or down-regulated (**b**) in ovarian cancer

Thus far, we have identified genetic alterations whose further confirmation may reveal possible diagnostic relevance. To test this, we analyzed the repercussions of these alterations in regard to the survival probability of ovarian cancer patients using different public transcriptomic datasets. Cox analysis of the mRNA levels of the upregulated genes showed that patients expressing high levels of *NOTCH3*, *CKAP4*, *PAX8* and *AP1M2* had a significantly shorter survival than patients with low expression levels of these genes. We found the same trend for *DUSP1* and *C-KIT*, but with no statistical significance (Fig. 1a).

On the other hand, the analysis of the downregulated genes showed that low levels of *BTG2*, *DUSP4*, *FBXL7*, *ST13* and *ST7L* were significantly associated with a shorter survival of the patients compared to those expressing high levels of these genes (Fig. 1b). Similarly, low expression levels of *ADRB3*, *RAD51*, *ANG* and *ESD* showed a clear tendency toward shorter survival but without statistical significance (Fig. 1b).

Finally, we analyzed all deregulated genes in combination and generated high- and low-risk groups with the Cox regression analysis. We used mRNA expression levels in the 4 databases with the highest numbers of patients of those analyzed above. The resulting Kaplan-Meier curves showed that the high-risk combinations of these 15 genes were clearly predictive of a worse prognosis (Additional file 1: Figure S2A). In addition, the expression levels of the genes of study in those high and low risk groups followed in general the same trend than those in the individual analyses. The analysis of the different stages also showed a clear and significantly lower survival probability for patients with the high-risk gene combination in all stages of the TCGA ovarian cystadenocarcinoma cohort compared to that of the low-risk group (Additional file 1: Figure S2B).

### Clustering analysis of the identified genes related to ovarian resistance

Platinum-based chemotherapy constitutes the backbone of systemic treatment in ovarian cancer, and platinum resistance represents a major prognostic factor for shorter event-free latency and lower survival compared to platinum-sensitive disease (Additional file 1: Figure S3). Therefore, the prognostic capabilities of the genes found in our meta-analysis, as well as the pathways involved, will be likely to predict the response to platinum therapies in ovarian tumors.

The identified genes are associated with a broad spectrum of activities (Table 1), and it is difficult to assign common functions that may be related to the phenotype of therapy resistance. To shed light on the pathways involved in platinum resistance, we used a network-based approach, overlaying the information from the differential gene expression analysis onto protein interaction networks and subnetworks using the string interaction network portal (https://string-db.org).

Most of the grouping analyses with all genes recognized DNA damage and repair pathways, based only on the *Rad51* gene (Additional file 1: Figure S4A), or the Adaptor-related protein complex 1 group of genes, based only on *AP1M2.* Other clusters around group 1, which contained the rest of the genes, were always grouped into three or four main clusters. The NOTCH pathway and the MAPK/MEK pathway, involving C-KIT, which was either related or not to the AKT/pathway, were found in all network combinations (Additional file 1: Figure S4B). On the other hand, some network combinations formed a cluster around Yamanaka’s factors (*NANOG*, *SOX2*, *KLF4* and *POU5F1)*, which were connected through *MYC*, *STAT3*, *NOTCH3* and *C-KIT* (Additional file 1: Figure S4C). Other genes, such as *ASD, FBL7, ST7L* or *ADRB,* were not significantly connected to the main clusters in most of the combinations. Although this analysis was not conclusive, these results may indicate that the DNA damage, MAPK/AKT (involving C-KIT), and the NOTCH pathways are recurrent, major networks that formed with the combination of the alterations found in our screenings. In addition, these networks have been repeatedly reported to support the CSC phenotype in ovarian tumors [16–22].

The previous data indicate two possible experimental hypotheses: first, the genes found in our screening may be altered in cultures that are enriched for CSCs and may therefore be markers of CSC enrichment in ovarian tumors; second, these “core” networks, the MAPK/AKT (involving C-KIT), NOTCH and DNA damage pathways, may be ideal therapeutic targets to recover platinum sensitivity. We will address both hypotheses in the following experiments.

### Relevance of CSCs in resistance to platinum

**Fig. 2:**
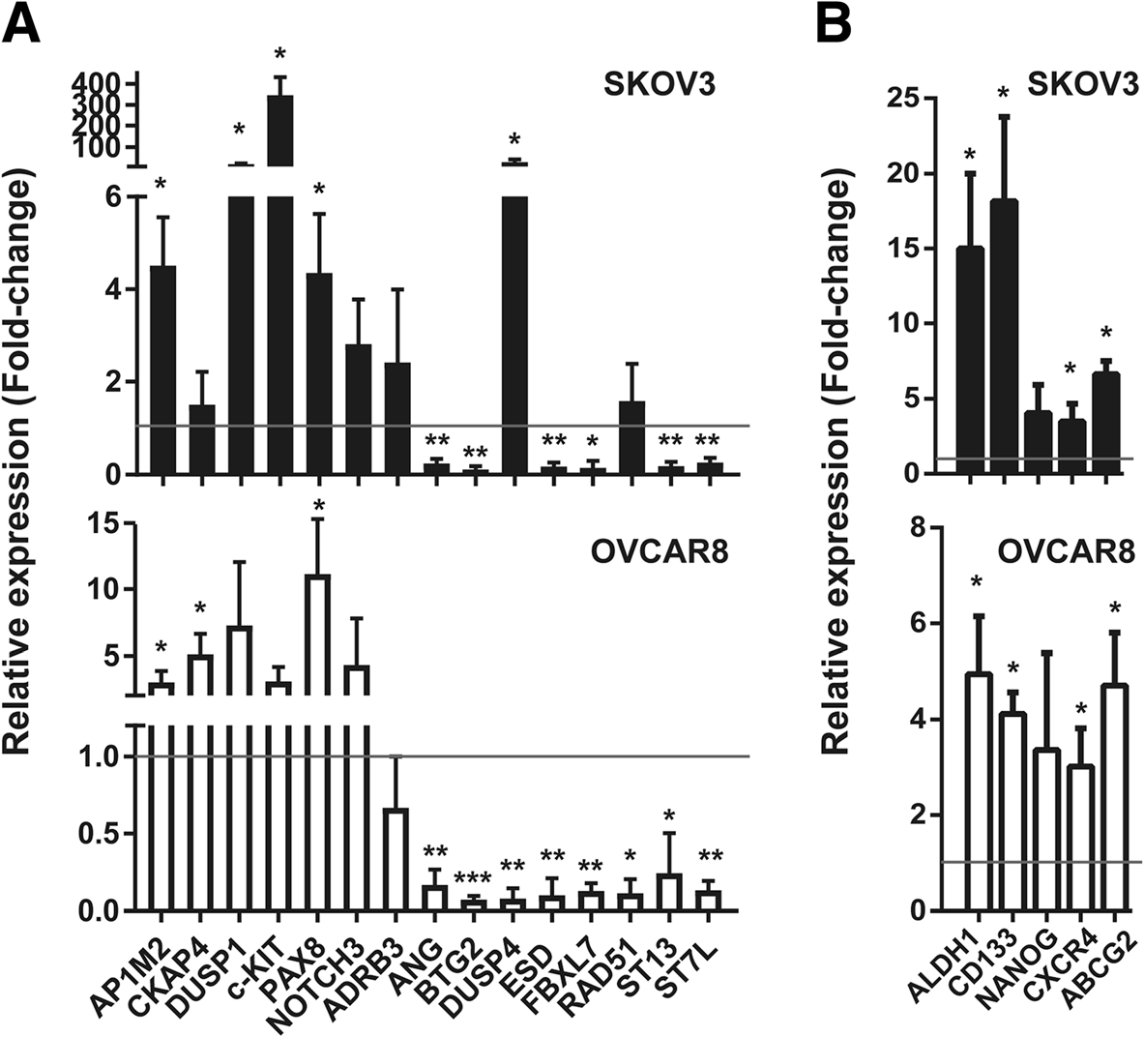
Gene expression analyses in tumorspheres from ovarian cancer cell lines. **a** Gene expression analyses by RT-qPCR of up- and down-regulated genes in tumorspheres and total adherent cell culture (total culture) samples from SKOV3 and OVCAR8 ovarian cancer cell lines. **b** Gene expression analyses by RT-qPCR of *C-KIT*, *CD133, NANOG*, *CXCR4* and *ABCG2* in tumorspheres and total culture samples from SKOV3 and OVCAR8 ovarian cancer cell lines. For (**a**) and (**b**), normalized expression values of tumorspheres compared to total culture samples (line at value 1) are shown. The average and SD of three independent experiments are shown. Statistical significance was assessed using the one-sample *t-*test. *, *P* < 0.05; **, *P* < 0.01; ***, *P* < 0.001

The existence of ovarian CSCs were reported more than 10 years ago, and ovarian CSCs have been reported to be responsible for resistance to chemo- and radiotherapy [17, 20, 23–26]. Based on these reported data, we explored whether cancer stem cells (CSCs) were enriched for our newly identified gene set. For this purpose, we seeded tumorspheres, which are supposed to be enriched in CSCs [22, 27–31], and compared their gene expression to that of mature cells from tumor cell lines in culture. To this end, we seeded cells from the ovarian cancer cell lines SKOV3 and OVCAR8 in CSC media and in low attachment plates to form tumorspheres. Once the tumorspheres had formed, we extracted RNA from the total adherent cell culture and from tumorspheres. We observed that the expression of the genes *AP1M2, CKAP4, DUSP1, c-KIT, PAX8* and *NOTCH3* shows a statistically significant increase in expression in tumorspheres from at least one of the cell lines (Fig. 2a**;** Additional file 1: Figure S5A). On the other hand, *ANG, BTG2, ESD, FBXL7, ST13* and *ST17L* are down-regulated in tumorspheres from both cell lines, while *DUSP4* and *RAD51* are only down-regulated in those from OVCAR8 cells. These data suggest that the expression of genes related to resistance in ovarian tumors are related to the stemness phenotype.

Then, as a control, we evaluated the expression of the CSC-related genes in tumorspheres. We observed, compared to the levels expressed by the adherent cell lines, that tumorspheres generated from both cell lines expressed significantly higher levels of *ALDH1*, *CD133*, *NANOG*, *CXCR4* and *ABCG2* (Fig. 2b**;** Additional file 1: Figure S5B), which have been proposed to be markers of ovarian CSC population [17, 20, 23–26].

### Predictive value for the response to cisplatinum

The data so far suggested that the stemness acquired by tumors is ultimately responsible for therapeutic resistance in ovarian tumors [16–22]. To confirm this hypothesis, we examined the expression of stemness-related genes in primary tumor samples. For this study, we used samples from platinum-resistant and platinum-sensitive ovarian tumors.

Hence, we sought to determine whether the identified genes could predict resistance to platinum drugs in primary samples from resistant tumors. Samples were obtained from the biopsies of ovarian cancer patients who underwent platinum-based chemotherapy, and the tumor response was assessed. Nonresponding subjects were identified, and the tumors were analyzed. A few nontumor samples from ovaries were also obtained and included as controls (Additional file 1: Table S3).

**Fig. 3:**
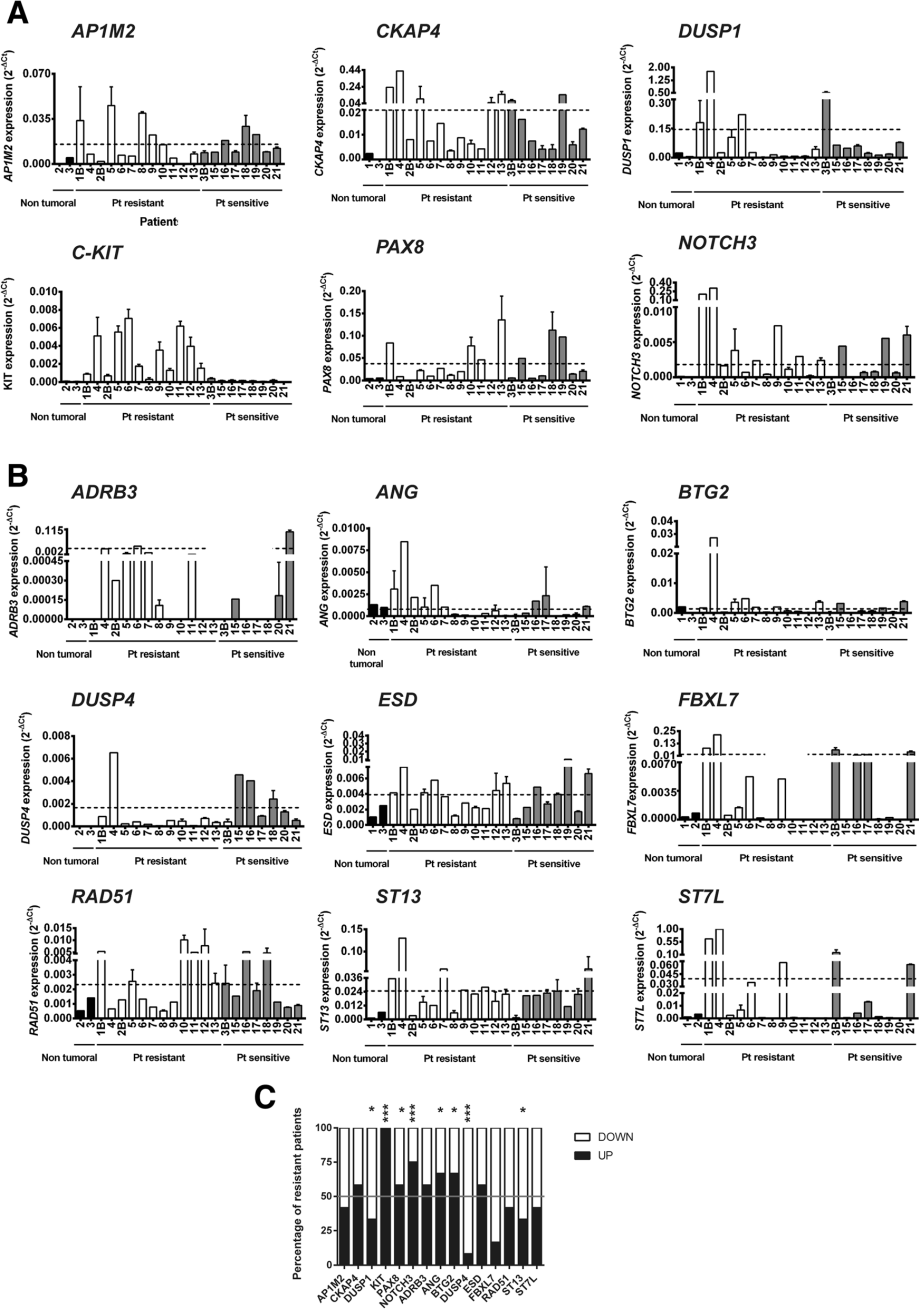
Gene expression analyses and platinum resistance in tumoral samples from ovarian cancer patients. **a** Gene expression analyses by RT-qPCR of the candidate up-regulated genes in non-tumoral and tumoral samples from ovarian cancer patients, resistant or sensitive to platinum treatment. **b** Same analysis as in (**a**) with the down-regulated genes in ovarian cancer. For (**a**) and (**b**), the average and SD of three independent experiments are shown. **c** Proportion of platinum-resistant patients with expression levels for each gene above (Up) or below (Down) the median value of the sensitive ones. Statistical significance was assessed using the Fisher’s exact test by comparison with the proportions of sensitive patients. *, *P* < 0.05, ***, *P* < 0.001

The analysis of gene expression showed that, at a global level, *c-KIT* was significantly up-regulated in resistant vs. sensitive patients, while *DUSP4* was significantly down-regulated (Fig. 3a-b**;** Additional file 1: Figure S6). Analysis of individual patients showed a number of them whose expression was also de-regulated, such as *AP1M2*, *PAX8, C-KIT* and *NOTCH3*. These genes showed very low levels of expression in non-tumor and platinum-sensitive tumor tissue, and a large increase in the expression levels was observed in some resistant tumors. For the down-regulated genes, high decreases in the expression were also observed in some individual patients for *ADRB3*, *FBXL7*, *RAD51*, *ST13* and *ST7L* (Fig. 3b**;** Additional file 1: Figure S6). However, a high heterogeneity was observed among the analyzed genes, and it was difficult to assign a specific “resistance” effect to the different genes (Fig. 3a), both for the up- and down-regulated ones. It is worth noting that the analysis of the proportion of resistant patients showing expression levels above or under the median value of the sensitive ones showed significant alterations of this proportion for *DUSP1*, *c-KIT*, *NOTCH3*, *ANG*, *BTG2R*, *DUSP4*, *FBXL7* and *ST13* (Fig. 3c), indicating that they are commonly de-regulated in platinum-resistant patients.

**Fig. 4:**
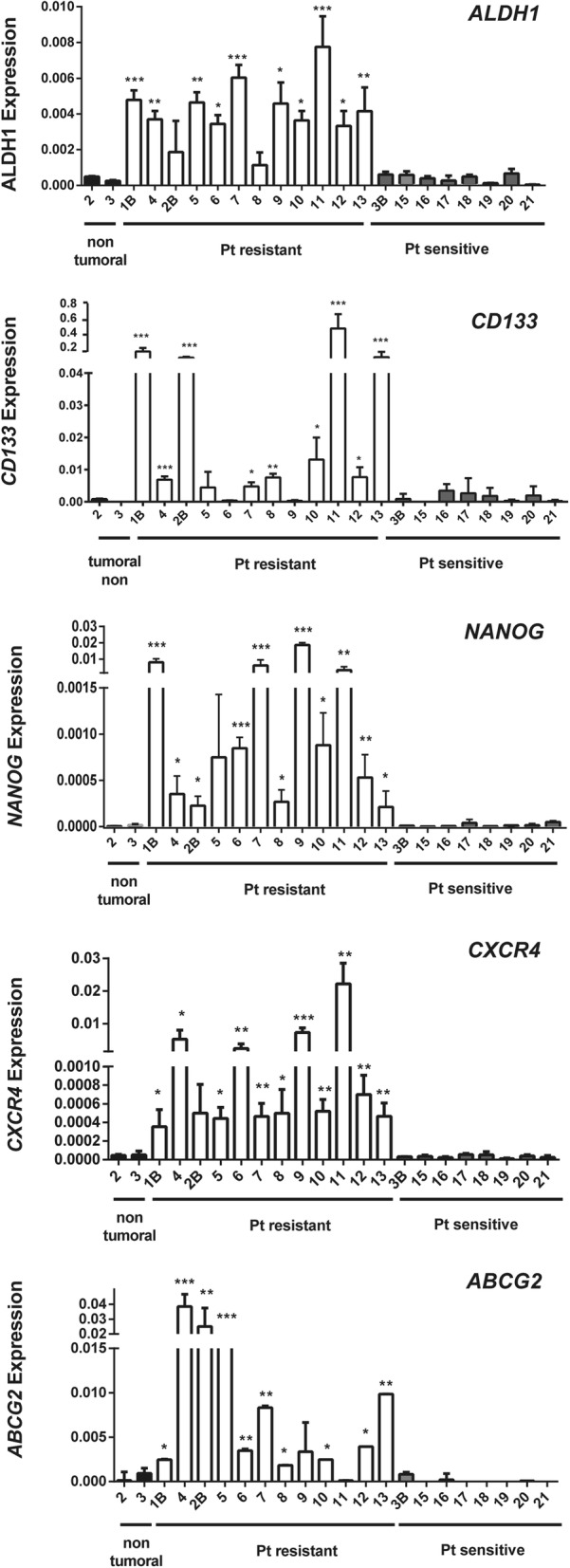
Gene expression analyses and platinum resistance in tumoral samples from ovarian cancer patients. Gene expression analyses by RT-qPCR of *C-KIT*, *CD133, NANOG*, *CXCR4* and *ABCG2* in non-tumoral and tumoral samples from ovarian cancer patients, resistant or sensitive to platinum treatment. The average and SD of three independent experiments are shown. Statistical significance was assessed using the Student’s *t-*test. *, *P* < 0.05; **, *P* < 0.01; ***, *P* < 0.001

In this platinum (Pt)-resistant vs Pt-sensitive cohort, we measured the levels of the CSC-related genes *ALDH1, CD133*, *ABCG2*, *CXCR4* and *NANOG*. We found that the Pt-resistant tumors expressed higher levels of these genes than the Pt-sensitive tumors (Fig. 4), indicating that the stemness capacity was higher in the Pt-resistant tumors than in the Pt-sensitive tumors. In addition, we also observed a large amount of heterogeneity among the different resistant samples (Fig. 4), possibly indicating that the resistance was provided by different redundant pathways or by different pools of CSCs with different characteristics.

### Sensitization to platinum drugs by inhibiting cancer stemness-related pathways

**Fig. 5:**
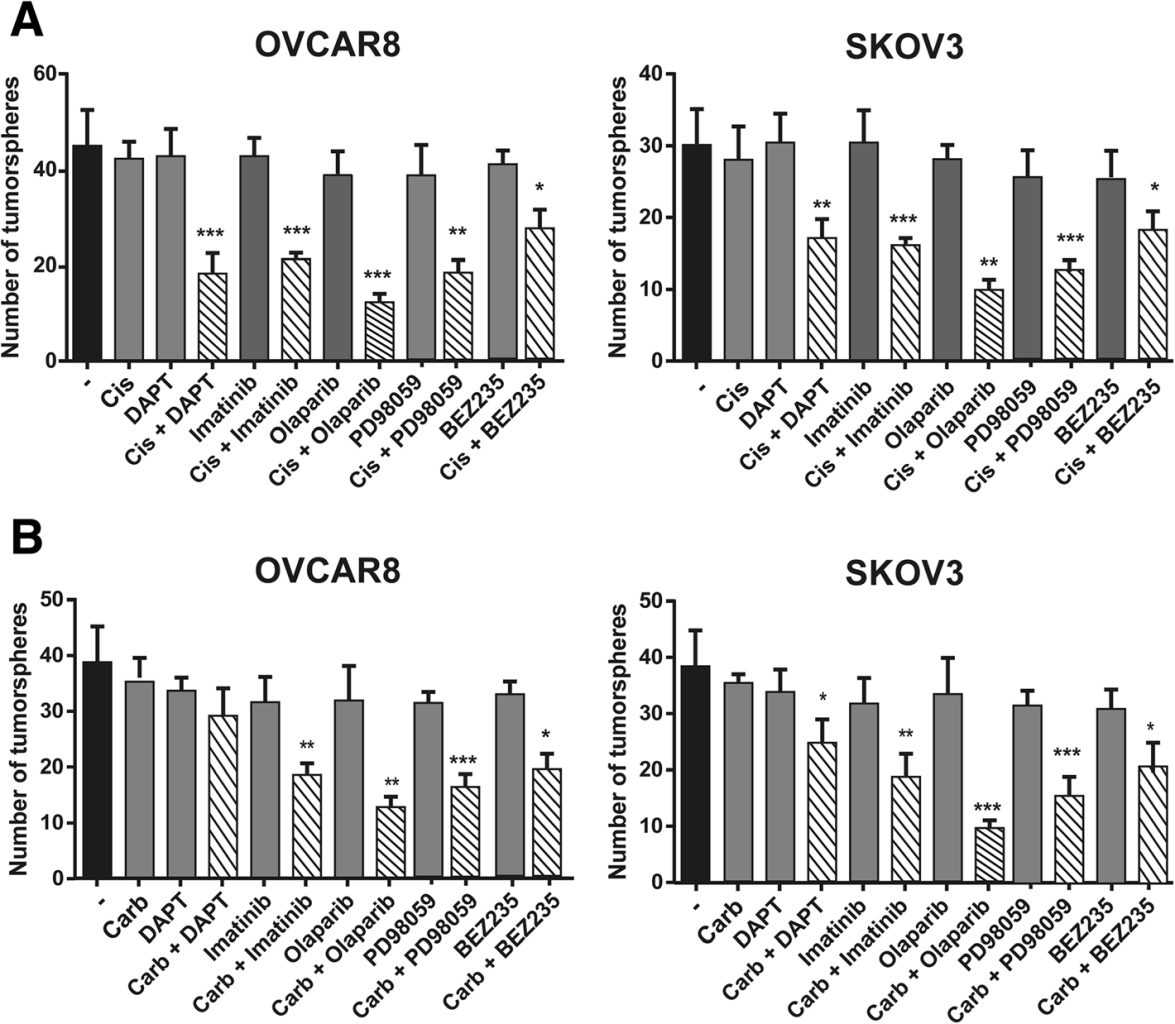
Notch, C-KIT, MAPK, PI3K and PARP inhibition reduces tumorsphere formation in ovary tumor cells. **a** Tumorsphere formation in OVCAR8 and SKOV3 cells treated with cis-platin (IC30; 0.3 μM), with or without gamma-secretase inhibitor DAPT (5 μM), tyrosine kinase inhibitor Imatinib (8 μM), PARP inhibitor olaparib (10 μM), MAPK inhibitor PD98059 (15 μM) or PI3K inhibitor BEZ235 (10 μM). **b** Tumorsphere formation in OVCAR8 and SKOV3 cells treated with carboplatin (IC30; 0.3 μM), with or without the inhibitors described in (A). The average and SD of at least three independent experiments are shown. Statistical significance was assessed using the Student’s *t*-test. *, *P* < 0.05; **, *P* < 0.01; ***, *P* < 0.001

Since resistance is dependent on the CSC phenotype, we hypothesized that the inhibition of these CSC networks might sensitize ovarian cancer cells to platinum treatments. To confirm this hypothesis, we seeded both of the tumor ovarian cell lines to form tumorspheres and treated them with suboptimal doses of cisplatin (IC30; 0.3 μM) or carboplatin (IC30; 0.3 μM) (Fig. 5 and Additional file 1: Figure S7) alone or in combination with suboptimal doses of inhibitors of the stem cell-related genes from our profile (Additional file 1: Table S4), such as DAPT (5 μM), a *NOTCH* inhibitor whose target is γ-secretase, imatinib (8 μM), a *C-KIT* inhibitor, PD98059 (15 μM), a MAPK inhibitor, and BEZ235 (10 μM), a PI3K inhibitor. We also treated the tumorspheres with platinum in combination with the PARP inhibitor olaparib (10 μM). In all cases, we used concentrations that did not reduce significantly the number or the size of the tumorspheres (Fig. 5).

We observed that the used doses of cis- or carboplatinum alone did not affect the survival or the number of tumorspheres, as with each of the targeted inhibitors alone (Fig. 5). However, treatment with the targeted inhibitors greatly reduced the number of tumorspheres in both cell lines when they were used in combination with cisplatin or carboplatin (Fig. 5) compared to that of the controls. In all cases, the combinations provided a significant reduction of approximately 50% in the number of tumorspheres compared to controls. Furthermore, the inhibition of the DNA damage pathway was slightly higher with the combination with both platinums compared to that of the other inhibitors, while DAPT, a NOTCH inhibitor, had lower activity in combination with carboplatin compared to the other inhibitors.

In the end, these data suggest that combinations of platinum drugs with C-KIT, MAPK, PI3K or PARP inhibitors may be a suitable therapy to avoid recidiva or metastasis in ovarian tumors or for the treatment of platinum-resistant tumors.

## Discussion

The cytotoxic activity of platinum complexes produces DNA alterations and increases the oxidative levels in tumor cells. This cytotoxic activity causes intra- and interstrand crosslinks and the formation of DNA adducts, provoking conformational changes that impair DNA replication. On the other hand, the increase of ROS species may induce DNA and mitochondrial damage, leading to a decrease in ATP activity. In addition, platinum-derived compounds produce alterations in cellular transport. Therefore, genetic events that alter any of these mechanisms may limit the efficacy of platinum compounds. Many mutations or alterations of the methylation profiles and epigenetic signals are involved in platinum resistance (TCGA database, cBioportal) [10–13, 32]. In addition, a recent analysis of a large number of patients with high-grade serous ovarian tumors showed a high degree of complexity, a high number of genomic aberrations and genetic alterations, and high levels of intra- and intertumoral heterogeneity [33–35]. Hence, the use of these alterations, most of them occurring with a very low frequency, as biomarkers to predict sensitivity or resistance to platinum-derived compounds is not currently useful. Since resistance to platinum treatments is one of the main causes of poor survival among ovarian cancer patients, the identification of prognostic and predictive biomarkers, as well as the understanding of the mechanisms driving resistance, is urgently needed.

We identified transcriptional targets that are possibly the common endpoints of the genetic alterations that are linked to platinum resistance in ovarian tumors. We found 15 genes that were transcriptionally altered, 6 of which were overexpressed and 9 of which were downregulated, belonging to different families. Compared to that in normal tissue, *CKAP4*, *DUSP1*, *PAX8*, *NOTCH3*, *C-KIT* and *AP1M2* were upregulated in tumor tissue, while *ADRB3*, *ANG*, *BTG2*, *ESD*, *ST13*, *ST7L*, *RAD51*, *DUSP4* and *FBXL7* were highly downregulated in tumor tissue. Individually, most of these genes showed prognostic value in terms of overall survival in ovarian cancer patients, where platinum-derived compounds are still the main therapy. The genes that did not show statistically significant differences still showed a clear trend in our analysis. On the other hand, the complete profile of all of the genes also showed a clear predictive capability for the prognosis of overall survival or relapse-free survival, independent of the tumor stage. This profile itself could be used to stratify patients due to its predictive value for platinum resistance. It would also be interesting to combine our profile with other clinical predictors, such as *CA125*, stage, histological type or the degree of differentiation, to provide an accurate clinical assessment with increased prognostic and predictive value that could help to stratify patients in clinical practice.

Numerous attempts have been made to identify signatures associated with ovarian cancer therapy resistance [16, 33–41]. Most of the multiple signature limitations involve the number of cases, representability of the different tumor types, further analysis of treatments, etc. A few of these signatures underwent further gene regulatory network analysis, identifying a unique network of genes that may potentially support current clinical practice, similar to our study. For example, Chudasama et al. found two proteins, RAD51AP1 and FSTL1, that were significantly overexpressed in ovarian cancer samples [36]. In our study, we observed the downregulation of *RAD51* in tumor samples, which may account for the RAD51AP1 increase. Liu et al. reported a list of 21 genes from a literature search that may be involved in ovarian cancer drug resistance [33–35]. One of these genes was *NOTCH3*, which was also identified in our analysis. Interestingly, 8 of the genes from that study are a part of our network (*FOS, JUN, BCL2, KRAS, MAPK1, MYC, NOTCH3* and *STAT3*), and most of the other genes are directly related to genes with similar functions to those found in our network (Bad and Bax are related to BCL2; EGFR and ERBB2 are receptor Ser/thr kinases that are related to Ras and the MAPK pathway; and Src, PIK3CA and AKT are also related to the Ras/MAPK pathway). It is important to take into account that most of the markers that have been proposed were related to the molecular mode of action of platinum, either preceding binding, directly related to the formation of adducts or related to the activation of the signaling pathway that is induced by DNA damage [11]. A small number of markers represented off-target effects that are not related to the mechanism of action of platinum at any level; only one of our 15 markers may be directly related to the DNA signaling pathways, *RAD51*, while the rest represent off-target effects. Interestingly, the MAPK pathway is at the core of our network and has been repeatedly described in the literature [10, 11, 33–35]; therefore, the MAPK pathway may represent one of the main targets that could be leveraged to overcome platinum resistance. Our data on the cytotoxicity of suboptimal doses of the treatments on the tumorspheres suggest that combinations of platinum drugs with C-KIT, MAPK, PI3K or DNA-damage inhibitors may be a suitable therapeutic strategy to increase activity, avoid recidiva or the metastasis of platinum-resistant ovarian tumors.

We confirmed that the transcriptional levels of the genes found in our meta-analysis were also altered in samples from patients with platinum-resistant tumors from our own patient cohort, reinforcing their value as prognostic markers. Furthermore, we also found increased levels of CSC markers in the samples from platinum-resistant patients, connecting the resistance to platinum treatment to the CSC phenotype. We think that the CSC phenotype also has a clear prognostic value. The analysis of the CSC-related genes that were identified in our work (*ALDH1*, *CD133*, *NANOG*, *CXCR4* and *ABCG2*) showed that outside of their relevance for defining the CSC phenotype, these genes have no relevant prognostic value, either alone or as a group (data not shown). This lack of prognostic value is probably due to the large amount of heterogeneity that was observed in the expression of these genes in human tumors. However, our profile of 15 genes, which are not mechanistically related to the mechanism of action of cisplatin and are related to the off-target resistance signaling pathways [11], have a clear prognostic ability to identify patients with ovarian tumors who are likely to develop resistance to platinum therapy.

Network analysis of these genes showed that most of the genes are integrated into 4 main clusters, three of which are linked directly to stemness. These networks are the NOTCH network, the MEK/MAPK network and, especially, the Yamanaka core (*NANOG*, *SOX2*, *OCT4* and *KLF4*). These networks are connected through *C-KIT*, *STAT3*, *NOTCH1* and *MYC*. These 4 genes lie at the middle of the three stemness networks and may be essential nodes to explore for therapeutic interventions.

Multiple markers, such as the Hoechst side population, CD133+, CD117 (c-KIT)+, ALDH1+ or CD44+ cells, have been described and used to identify CSCs from ovarian tumors [16–22]. However, the concept of the ovarian CSC is controversial and has not been properly demonstrated. A unique CSC population has not been described, and it has not been concluded whether CSCs are responsible for ovarian cancer resistance. Some markers, such as CD133+, CXCR4+ [42] or ALDH1 + CD133+ [21], also define different CSC populations in ovarian tumors. In some cases, as in CD133+/CXCR4+ cells, these populations are also related to an increased expression of the stemness transcriptional core, *SOX2*, *OCT4*, *KLF4* and *NANOG* [42]. It has also been suggested that the expression of some of these markers depends on the environmental conditions [21]. These data agree with the apparent heterogeneity of the CSC markers that was present in different resistant tumors, which probably reflects the selection of one of the different subpopulations, but each of these markers are related to the physiology of CSCs [43–49]. However, a complete demonstration has not been provided. Our work directly analyzed 15 genes that were identified with transcriptional screening, 3 of which are directly related to stemness (*NOTCH3*, *C-KIT* and *PAX8*) and 5 of which were direct markers of CSCs (*ALDH1*, *ABCG2*, *CXCR4*, *CD133* and *NANOG*) in ovarian tumor samples from platinum-resistant patients, corroborating this relationship. All of these data suggest that these CSC markers are not mutually exclusive and that they may appear as a result of multiple genetic changes that occur during the process of tumorigenesis.

The heterogeneity of the different CSC markers found among the resistant tumors was remarkable (Fig. 4). The correlation among the different markers indicated that different CSC populations may have been present. Most of the CSC markers correlated with *ALDH1*, and we also found some correlation between *CXCR4* and *CD133* (Additional file 1: Figure S8), suggesting a general CSC population was represented by *ALDH1*, and that there was a CXCR4 + CD133 subpopulation, as has been previously reported [21, 42]. However, these trends also presented some heterogeneity. This may indicate that resistance is provided by different redundant pathways or by different pools of CSCs, most likely with distinct characteristics. Whether these pathways or CSC pools have different features and how this translates into the heterogeneity of the tumors needs to be further explored.

As previously mentioned, the networks that we identified are connected through *C-KIT/MAPK/AKT* or *NOTCH*. These genes lie at the middle of the networks and may be essential nodes that could be used to explore novel therapeutic interventions. In fact, we tested whether their inhibition produced any effect on CSC resistance. Our data showed that the inhibition of any of these nodes at suboptimal doses in combination with cisplatin or carboplatin significantly reduced the growth of the tumorspheres, elements that are enriched for CSCs. On the other hand, we expect much better results upon the inhibition of more than one node of the pathway, and this should preferably be evaluated in vivo; however, this hypothesis remains to be tested.

## Conclusions

We have identified transcriptional targets that may be the common endpoints of genetic alterations that are linked to platinum resistance in ovarian tumors. The relative expression of these genes, either alone or in combination, could serve as biomarkers of prognosis and platinum resistance. In addition, the expression of these genes is connected to the CSC phenotype. Therefore, the measurement of CSC-related factors in ovarian tumors has emerged as a new method of detecting platinum resistance, even though the expression of the CSC markers is heterogeneous among the different resistant tumors. Finally, our results in vitro show that the inhibition of druggable CSC-related targets sensitizes CSC-enriched tumorspheres to platinum therapies, suggesting a new alternative for the treatment of patients with platinum-resistant ovarian cancer.

## Additional file

Additional file 1: **Table S1.** GSEs explored in this work. **Table S2.** Genetic data on cBioportal for all genes in the TCGA database (serous cystadenocarcinoma, *n* = 606) (ST7L, not found). **Table S3.** Patient Cohort characteristics. **Table S4.** Inhibitors tested in tumorspheres from ovarian cancer cell lines. **Figure S1.** Promoter methylation levels found in highly de-regulated genes in ovarian tumors. **Figure S2.** Analysis of the survival probability of ovarian cancer patients in the TCGA dataset by the expression of the grouped identified genes. **Figure S3.** Event free survival probability of ovarian cancer patients according to platinum sensitivity. **Figure S4.** Interaction networks of de-regulated genes in ovarian cancer. **Figure S5.** Gene expression analyses in tumorspheres from ovarian cancer cell lines. **Figure S6.** Box plots showing expression data of all patients from Fig. 3 grouped in sensitive (S) and resistant (R) to platinum therapy. **Figure S7.** Effects of platinum treatment in ovarian cancer cells. **Figure S8.** Correlations between CSC markers in platinum therapy sensitive and resistant ovarian cancer patients. (PDF 38000 kb)

## Data Availability

All data and materials are available upon request.

## Abbreviations

AMC: Academic Medical Center
CSC: Cancer Stem Cell
OC: Ovarian Cancer
Pt: Platinum
