# Supplementary material for: New markers for human ovarian cancer that link platinum resistance to the cancer stem cell phenotype and define new therapeutic combinations and diagnostic tools
Source: J Exp Clin Cancer Res. 2019 Jun 3;38:234. doi: 10.1186/s13046-019-1245-5 (PMC6547556; doi:10.1186/s13046-019-1245-5)
Supplement: Supplementary file 1 — Table S1. GSEs explored in this work. Table S2. Genetic data on cBioportal for all genes in the TCGA database (serous cystadenocarcinoma, n = 606) (ST7L, not found). Table S3. Patient Cohort characteristics. Table S4. Inhibitors tested in tumorspheres from ovarian cancer cell lines. Figure S1. Promoter methylation levels found in highly de-regulated genes in ovarian tumors. Figure S2. Analysis of the survival probability of ovarian cancer patients in the TCGA dataset by the expression of the grouped identified genes. Figure S3. Event free survival probability of ovarian cancer patients according to platinum sensitivity. Figure S4. Interaction networks of de-regulated genes in ovarian cancer. Figure S5. Gene expression analyses in tumorspheres from ovarian cancer cell lines. Figure S6. Box plots showing expression data of all patients from Fig. 3 grouped in sensitive (S) and resistant (R) to platinum therapy. Figure S7. Effects of platinum treatment in ovarian cancer cells. Figure S8. Correlations between CSC markers in platinum therapy sensitive and resistant ovarian cancer patients. (PDF 38000 kb) [file 13046_2019_1245_MOESM1_ESM.pdf]

Supplementary Table 1: GSEs explored in this work

GSE	n	Main Stages	Main represented subtypes
DUKE-OC	133	IIIC/IV	Serous ADC
GSE9891	278	III (85%)	Serous ADC
GSE17260	110	IIIB C/IV	Serous ADC
GSE26712	195	10 NT	
		185 T	Ov Carc
GSE14764	80	IIIA C	Serous ADC
GSE3208	23	10 NT	
		13 T	Ov Carc
GSE6008	103	4NT	
		99 T	Endometroid
			Serous ADC
GSE12470	53	10 NT	
		43 T	Serous ADC
Lu (CCR-2005)	50	5 NT	
		45 T	Ov Carc

n= number of samples

T= Tumor; NT= not tumor

Supplementary Table 2: Genetic data on cBioportal for all genes in the TCGA database (serous cystadenocarcinoma, n=606) (ST7L, not found)

**Gene**	**Overall alterations**	**% upregulating alterations ***	**% downregulating alterations ***
NOTCH3	20%	16 % Amplifications<br>20% mRNA upReg	
AP1M2	20%	11% Amplifications<br>15% mRNA upReg	6% mRNA DownR
c-KIT	8%	2 % Amplifications<br>4% mRNA upReg<br>2% protein UpReg	2% mRNA DownR
DUSP1	7%	2.5 % Amplifications<br>2.6% mRNA upReg	1.5% Deletions<br>0.5% mRNA DownR
PAX8	10%	5 % Amplifications	0.5% Deletions<br>4.5 % mRNA DownR
CKAP4	13%	2% Amplifications<br>3% mRNA upReg	8%% mRNA DownR
ANG	10%	4.5% Amplifications<br>4.5% mRNA upReg	1% mRNA DownR
ADRB3	7%	3.2 % Amplifications<br>2% mRNA upReg	1% Deletions<br>0.8% mRNA DownR
BTG2	12%	7.5% Amplifications<br>3.5% mRNA upReg	1% mRNA DownR
FBXL7	16%	11 % Amplifications<br>5 % mRNA upReg	1% mRNA DownR
RAD51	9%	2.7 % Protein UpReg<br>3 % mRNA UpReg	3% Deletions<br>0.3% Protein DownR

ST13	9%	1 % Amplifications	1.5 % Deletions<br>6.5 % mRNA DownR
DUSP4	7%	0.7 % Amplifications<br>1% mRNA upReg	5.3% Deletions
ESD	14%	1% mRNA upReg	2% Deletions<br>13% mRNA DownR

*Some alterations may coexist

Supplementary Table 3: Patient Cohort characteristics

	Sensitive N=9 (40%)	Resistant N=13(60%)
**Age (years)**<br>• Mean	 62,0	 51,0
**Stage (FIGO 2014)**<br>• Located disease (stage I)<br>• Advanced disease (Stage IIB-IV)	 2 (22%)<br>7 (88%)	 2 (15%)<br>11 (85%)
**Treatment**<br>• Carbo + Paclitaxel<br>• Carbo + Paclitaxel + beva<br>• Carbo monoterapia	 6 (68%)<br>3 (32%)<br>0	 12 (92%)<br>0<br>1 (8%)
**Progression disease after treatment**<br>• Yes<br>• No	 <br>6 (67%)<br>3 (33%)	 <br>13 (100%)<br>0
**Histology**<br>• Serous carcinoma<br>• Clear cell carcinoma<br>• Others	 7 (78%)<br>2 (22%)<br>0	 8 (62%)<br>3 (23%)<br>2 (15%)

Supplementary Table 4: Inhibitors tested in tumorspheres from ovarian cancer cell lines

**Inhibitors**	**Target**	**Doses**
DAPT	Gamma-secretase (NOTCH pathway)	5 mM
Imatinib	*cKIT*	8 mM
Olaparib	PARP	10 mM
PD98059	MAPK	15 mM
BEZ235	PI3K	10 mM

SUPPLEMENTARY FIGURE LEGENDS

Supplementary Figure S1. Promoter methylation levels found in highly de-regulated genes in ovarian tumors. Data shows the average levels of non-tumoral samples (normal) or ovarian tumors (cancer). Statistical significance was assessed using the Student's *t*-test. * ($p<0.05$), ** ($p<0.01$), *** ($p<0.001$).

Supplementary Figure S2. Analysis of the survival probability of ovarian cancer patients in the TCGA dataset by the expression of the grouped identified genes. (A) Cox analysis of survival probability of patients according to mean levels of mRNA of grouped 15 genes. Kaplan-Meier curves of the survival probability of the 15 genes grouped according to low or high risks in ovarian cancer using SurvExpress Tool (1) **(B)** Cox analysis of survival probability of patients according to mean levels of mRNA of grouped 15 genes. Kaplan-Meier curves of the survival probability of the 15 genes grouped according to low or high risks in different stages of ovarian cancer using SurvExpress Tool (1).

Supplementary Figure S3. Event free survival probability of ovarian cancer patients according to platinum sensitivity. In the database GSE63885 (2), patients were classified according to their disease-free survival (DFS): highly sensitive patients (DFS > 732 days); moderately sensitive patients (DFS between 180 and 732 days); resistant patients (DFS < 180 days).

Supplementary Figure S4. Interaction networks of de-regulated genes in ovarian cancer. (A) Rad51 protein interaction networks. **(B)** Notch, MAPK, RAS/AKT, KIT and AP1M protein interaction networks. **(C)** MAPK, Notch, AP1M and Stem cell core protein interaction networks.

Supplementary Figure S5. Gene expression analyses in tumorspheres from ovarian cancer cell lines. **(A)** Gene expression analyses by RT-qPCR of up- and down-regulated genes in tumorspheres and total adherent cell culture (total culture) samples from SKOV3 and OVCAR8 ovarian cancer cell lines. **(B)** Gene expression analyses by RT-qPCR of *C-KIT*, *CD133, NANOG*, *CXCR4* and *ABCG2* in tumorspheres and total culture samples from SKOV3 and OVCAR8 ovarian cancer cell lines. For (A) and (B), the average and SD of three independent experiments are shown. Statistical significance was assessed using the Student's *t*-test. *, $P < 0.05$; **, $P < 0.01$; ***.

Supplementary Figure S6. Box plots showing expression data of all patients from Figure 3 grouped in sensitive (S) and resistant (R) to platinum therapy. Average fold-change is shown on top. Statistical significance was assessed using the Student's *t*-test. *, $P < 0.05$; **, $P < 0.01$.

Supplementary Figure S7. Effects of platinum treatment in ovarian cancer cells. (A) Representative cytotoxicity experiment showing the average and SD of three biological replicates. The IC30 is the concentration of platinum necessary to induce 30% cell death. **(B)** Representative images of tumorspheres formed by OVCAR8 and SKOV3 cells treated with cis-platin or carboplatin

(IC30; 0.3 μM), with or without gamma-secretase inhibitor DAPT (5 μM), tyrosine kinase inhibitor Imatinib (8 μM), PARP inhibitor olaparib (10 μM), MAPK inhibitor PD98059 (15 μM) or PI3K inhibitor BEZ235 (10 μM). Scale bar: 100 µm.

Supplementary Figure S8. Correlations between CSC markers in platinum therapy sensitive and resistant ovarian cancer patients.

REFERENCES:

1. Aguirre-Gamboa R, Gomez-Rueda H, Martínez-Ledesma E, Martínez-Torteya A, Chacolla-Huaringa R, Rodriguez-Barrientos A, et al. SurvExpress: an online biomarker validation tool and database for cancer gene expression data using survival analysis. PLoS One. 2013 16;8(9):e74250.
2. Lisowska KM, Olbryt M, Dudaladava V, Pamuła-Piłat J, Kujawa K, Grzybowska E, Jarząb M, Student S, Rzepecka IK, Jarząb B, Kupryjańczyk J. Gene expression analysis in ovarian cancer - faults and hints from DNA microarray study. Front Oncol. 2014 4:6.

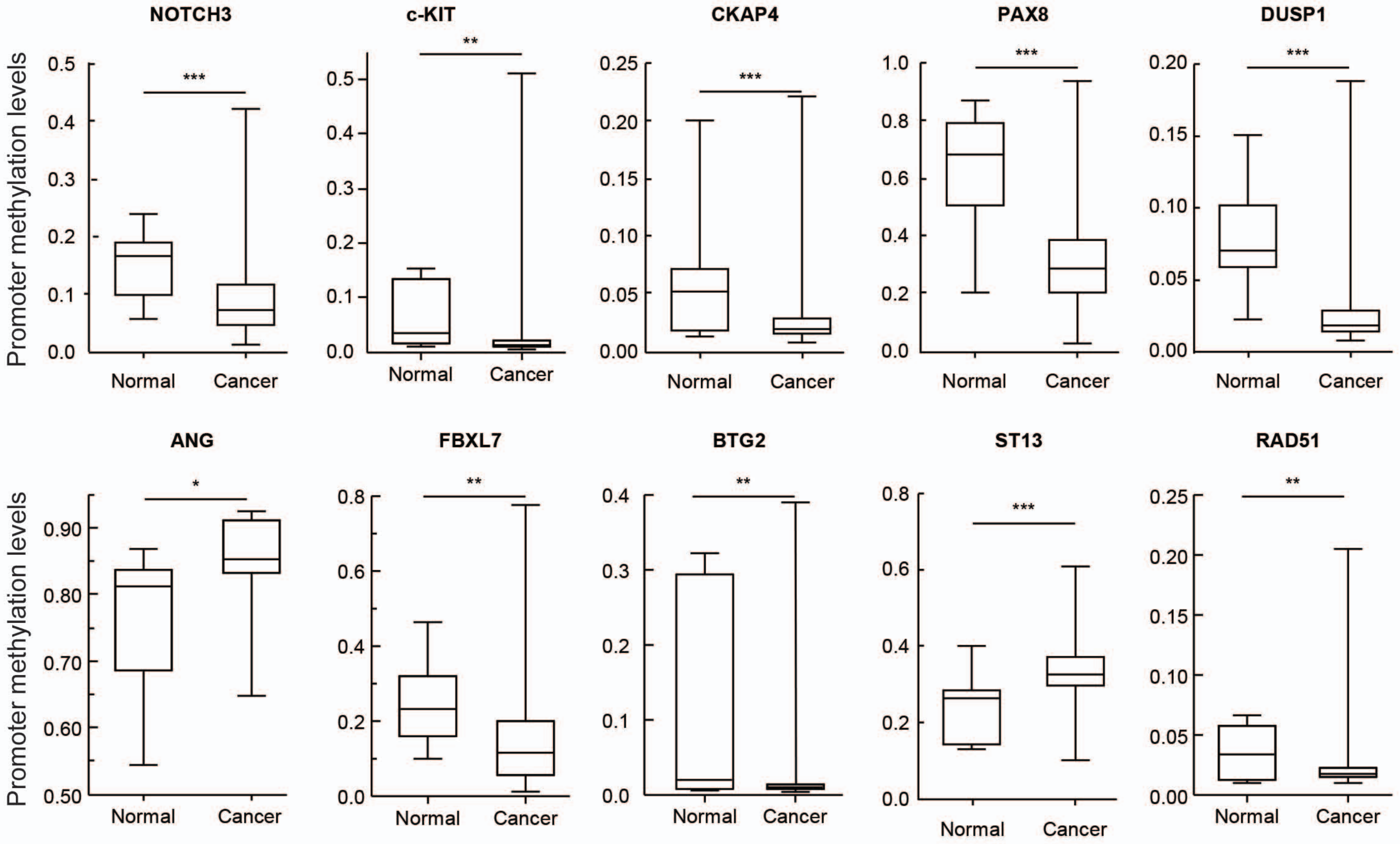

Supplementary Figure S1

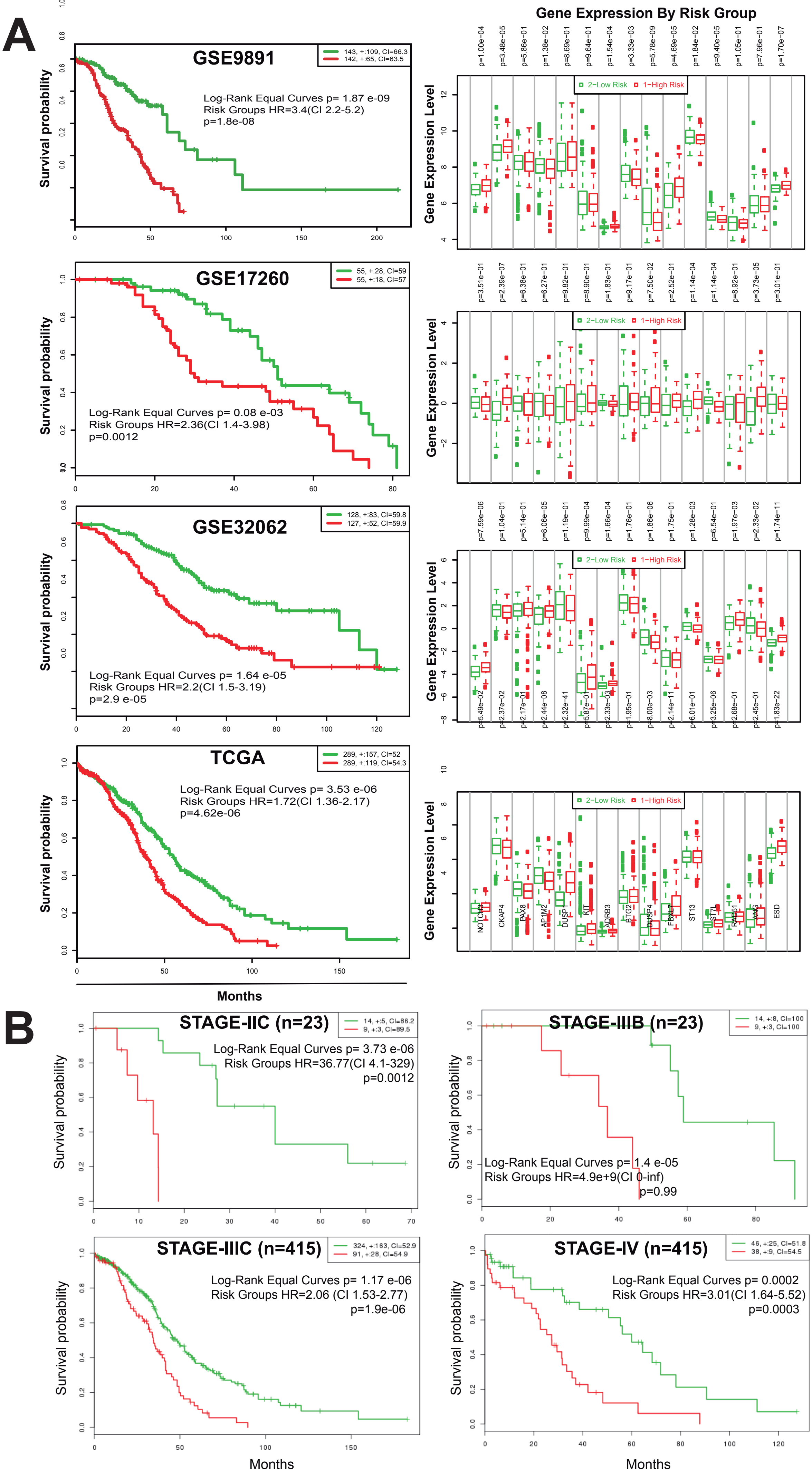

Supplementary Figure S2

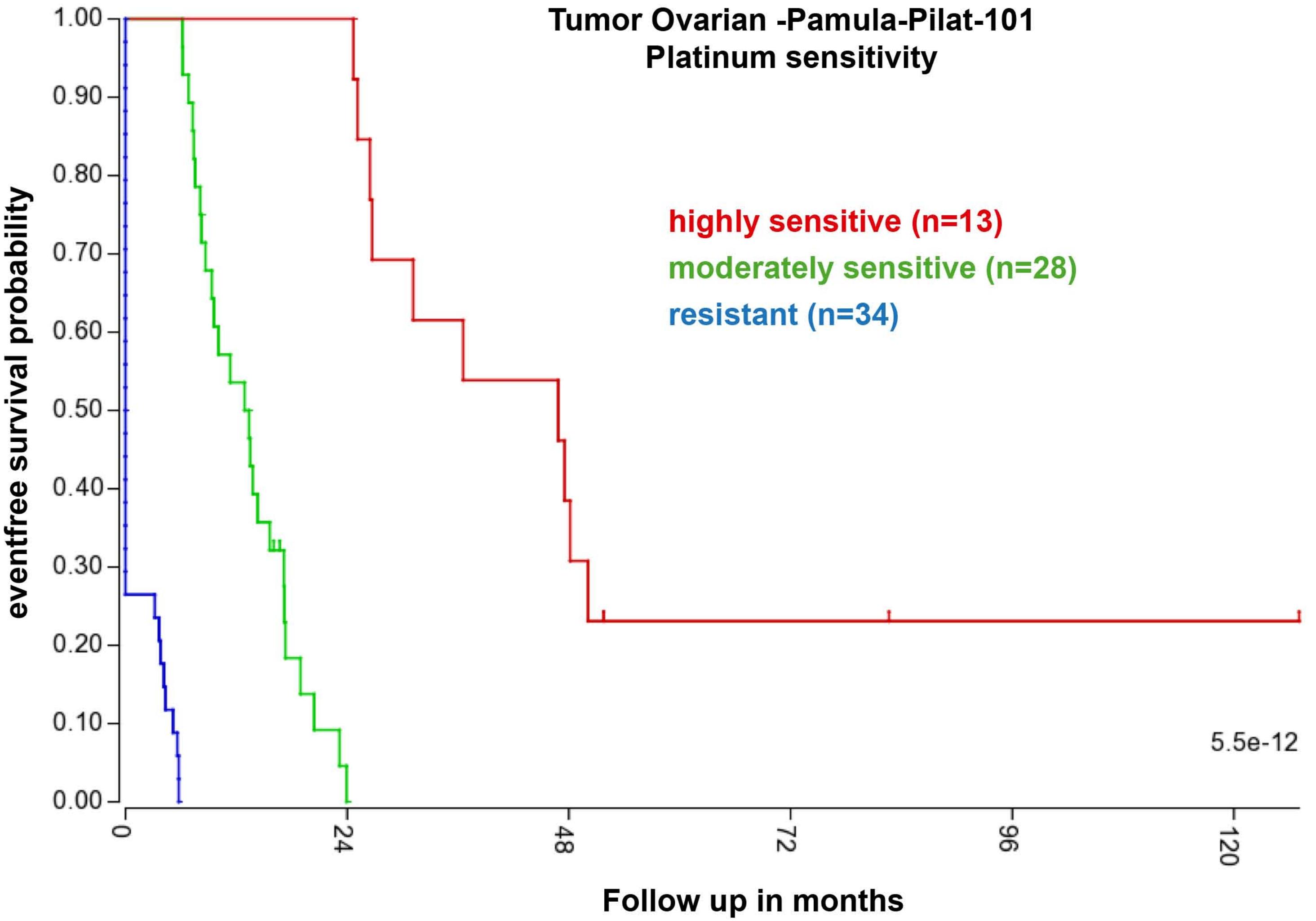

Supplementary Figure S3

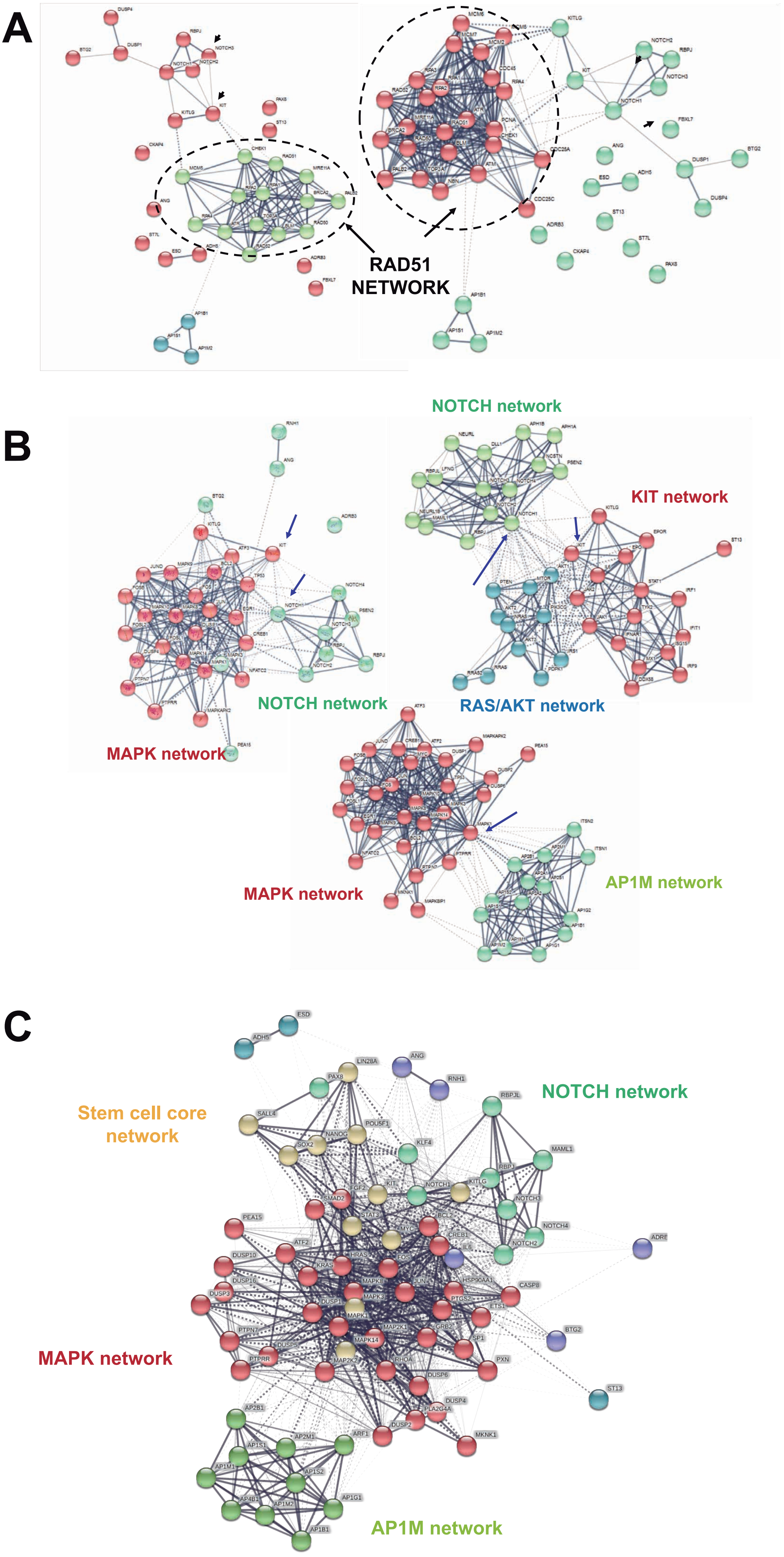

Supplementary Figure S4

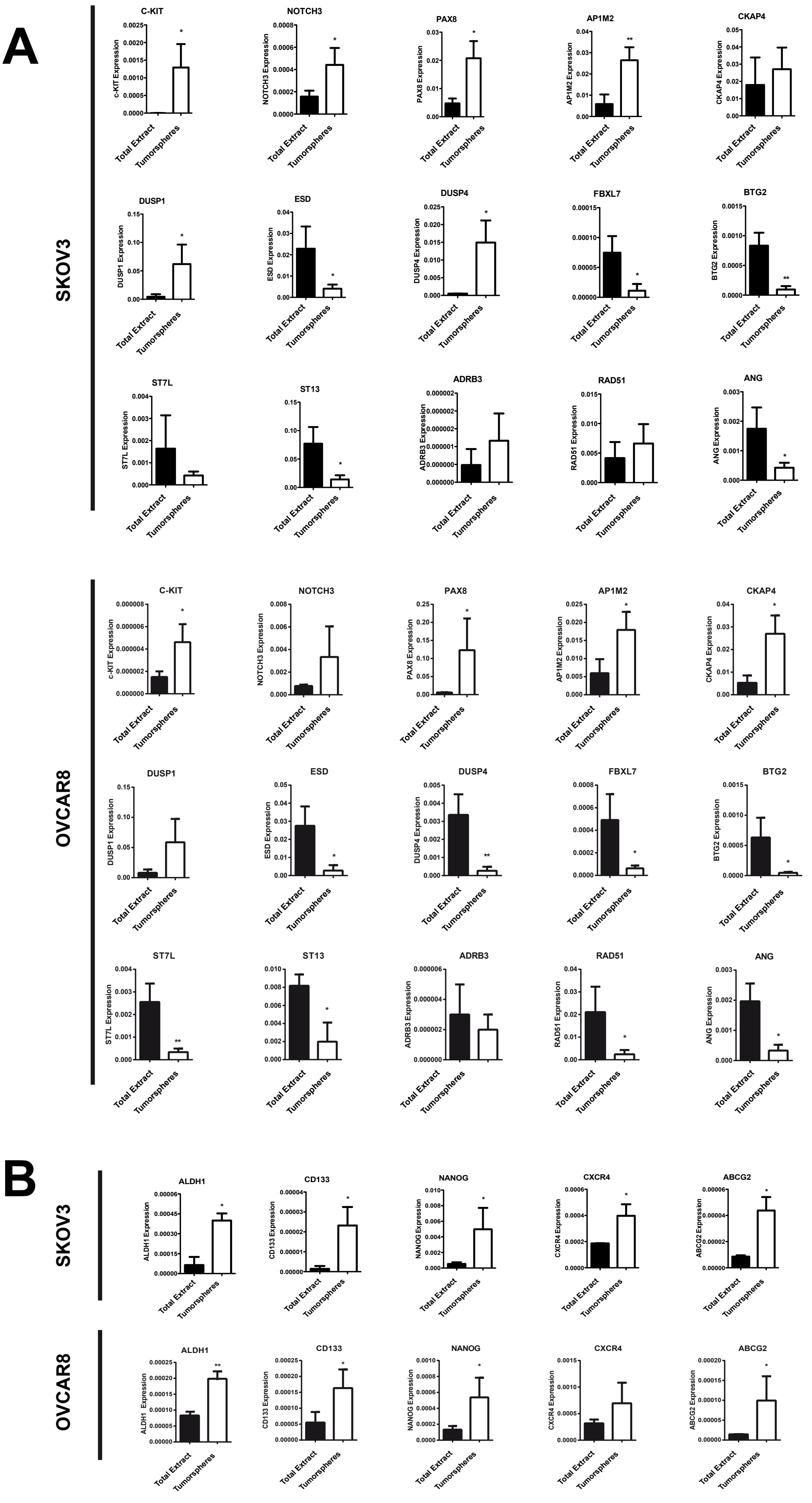

Supplementary Figure S5

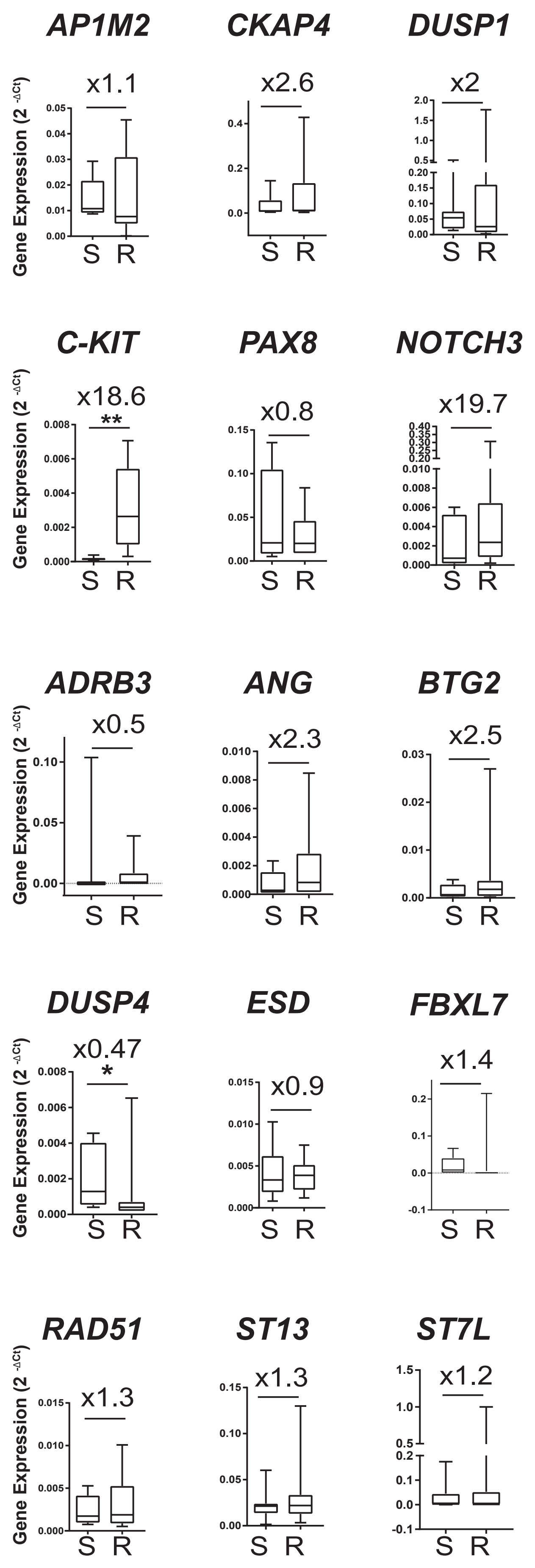

Supplementary Figure S6

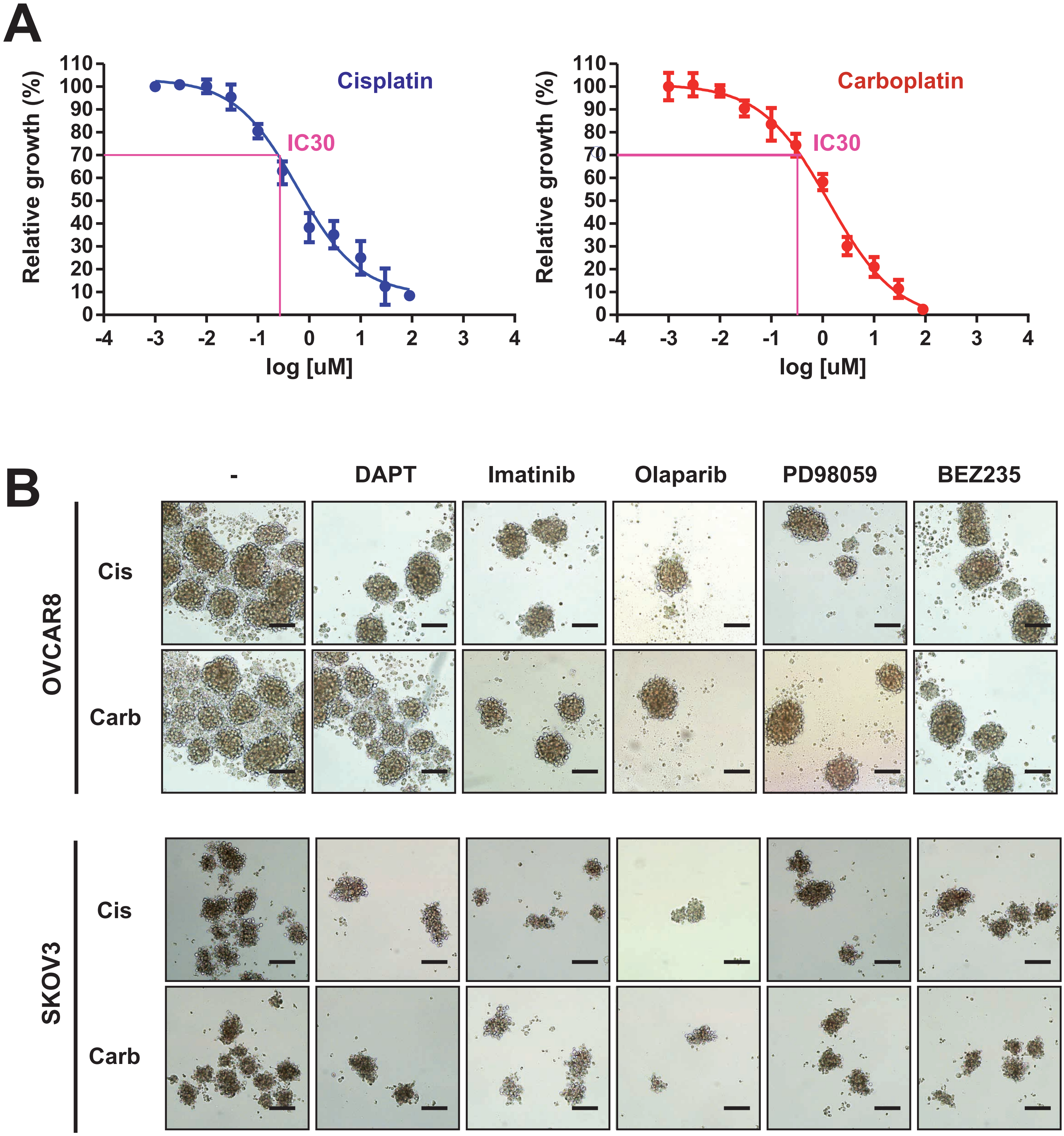

Supplementary Figure S7

	ABCG2		*ALDH1*		*CD133*		*CXCR4*		*NANOG*	
	S	R	S	R	S	R	S	R	S	R
ABCG2	1,00	1,00								
ALDH1	0,21	-0,22	1,00	1,00						
CD133	-0,33	-0,28	0,07	0,74	1,00	1,00				
CXCR4	-0,08	-0,17	-0,32	0,62	0,15	0,85	1,00	1,00		
NANOG	-0,42	-0,31	-0,09	0,57	-0,17	0,02	0,37	0,21	1,00	1,00

Supplementary Figure S8
